# Chikungunya Virus: What a Transfusion Service Needs to Know

**DOI:** 10.3390/v18080924

**Published:** 2026-08-21

**Authors:** Daniele Focosi, Giuseppe Sberna, Francesca Colavita, Fabrizio Maggi, Massimo Franchini

**Affiliations:** 1 North-Western Tuscany Blood Bank, Pisa University Hospital, 56126 Pisa, Italy; 2 Laboratory of Virology and Laboratories of Biosecurity, National Institute for Infectious Diseases “Lazzaro Spallanzani”–IRCCS, 00149 Rome, Italy; 3 Division of Hematology and Transfusion Medicine, “Carlo Poma” Hospital, 46100 Mantua, Italy

**Keywords:** chikungunya virus, arbovirus, transfusion, transplantation

## Abstract

*Togaviridae* represents a candidate family for emerging public health emergencies of international concern. Among them, chikungunya virus (CHKIV) has caused recurrent epidemics in the temperate zone of most continents and represents a threat to the safety of blood supplies. The global expansion of vectors (i.e., *Aedes* mosquitoes) and increasing international travel have contributed to the growing epidemiological importance of this pathogen. In fact, CHIKV has been reported in more than 110 countries across the following four continents: Africa, Asia, Americas, and Europe. Consequently, public health authorities have emphasized the importance of surveillance, vector control programs, outbreak preparedness, and blood safety measures. The aim of this review was to summarize the current knowledge of CHIKV considering virology, molecular epidemiology, transmission mechanisms, clinical features, preventive strategies, and blood safety implications, with particular attention to issues relevant to transfusion services.

## 1. Introduction

Chikungunya virus (CHIKV) is an emerging mosquito-borne arbovirus. The global expansion of vectors (i.e., *Aedes* mosquitoes) and increasing international travel have contributed to the growing epidemiological importance of this pathogen. In fact, CHIKV has been reported in more than 110 countries across the following four continents: Africa, Asia, Americas, and Europe [1,2,3].

CHIKV, also named *Alphavirus chikungunya*, belongs to the genus *Alphavirus*, family *Togaviridae* [4,5]. The genus *Alphavirus* includes not only CHIKV, but also different viruses that are associated with outbreaks, arthritogenic disease, or neuroinvasive infections, like Ross River virus (RRV), Mayaro virus (MAYV), O’nyong-nyong virus (ONNV), Sindbis virus (SINV), Venezuelan equine encephalitis virus (VEEV), and Eastern equine encephalitis virus (EEEV) [4,5].

CHIKV has become increasingly relevant following the establishment of *Aedes albopictus* in several regions and the occurrence of autochthonous outbreaks in countries previously considered non-endemic [6,7]. For example, in Europe outbreaks have become increasingly frequent and geographically widespread since 2004 [1,2,3]. Consequently, public health authorities have emphasized the importance of surveillance, vector control programs, outbreak preparedness, and blood safety measures [6,7].

The aim of this review was to summarize the current knowledge of CHIKV considering virology, molecular epidemiology, transmission mechanisms, clinical features, preventive strategies, and blood safety implications, with particular attention to issues relevant to transfusion services.

## 2. Virological Features

CHIKV genome is a single positive-stranded RNA of 11.8 kb in length consisting of 5′ and 3′ untranslated regions (UTRs) and the following two open reading frames: ORF1 encoding four nonstructural proteins of the viral replicase complex (nsP1–4, with nsP4 representing the RNA-dependent RNA polymerase) and ORF2 (encoded by a subgenomic RNA) encoding structural polyproteins C, E3, E2, 6K, and E1 [8,9,10]. CHIKV was isolated the first time during the 1953 outbreak in Tanzania [11,12]. CHIKV is phylogenetically classified into the following three major genotypes [13]: East/Central/South/African (ECSA), West African (WA), and Asian. In 2005, ECSA CHIKV genotype re-emerged and was later referred as the Indian Ocean Lineage (IOL). Factors associated with CHIKV transmission include environmental/ecological conditions, abundance of mosquito egg laying habitats, immunologically naive populations, and viral genetics/mutations. Attack rates may be explained by surveillance practices, season of CHIKV introduction into a country or a region, vector density and activity, vector control measures, or lifestyle differences. Previously, CHIKV was transmitted mainly through *Aedes aegypti* [14]. However, an alanine-to-valine mutation at position 226 within the E1 glycoprotein (E1-A226V) in IOL CHIKV was shown to increase viral replication in *Aedes albopictus* [15]. For this reason, IOL CHIKV might widely spread to where this mosquito is abundant, particularly Thailand, Singapore, Malaysia, Asia, and even in temperate climate countries such as Italy and France in Europe.

Considering the viral entry process, CHIKV exploits different receptors in different cell types [16,17,18,19,20,21,22]. While ATP synthase β subunit (ATPSβ) is a receptor in mosquito cells [16], in humans the major receptors are prohibitins (PHB1 and PHB2) [17], glycosaminoglycans [18,19], CD147/basigin/EMMPRIN [20], MXRA8 (a receptor shared with other arthritogenic *Alphaviruses*, including RRV, MAYV, and ONNV) [21], and phosphatidylserine (PtdSer)-mediated virus entry-enhancing receptors (PVEERs) (such as TIM-1, a receptor shared with Ebolavirus, and Axl) [19,22]. All these receptors bind the domain B of the viral E2 glycoprotein (and occasionally of the E1 glycoprotein), which is the target of infection-neutralizing antibodies. Notably, some enveloped viruses have evolved to incorporate PtdSer in the viral membrane, hence disguised as apoptotic bodies, a phenomenon termed as apoptotic mimicry [23].

After binding to a receptor on the plasma membrane, CHIKV enters human cells primarily by clathrin-mediated endocytosis and membrane fusion [24], although clathrin-independent pathways have also been identified [25].

## 3. Clinical Manifestations, Outcomes, and Diagnosis

Chikungunya disease is an acute febrile illness [1,2,26,27,28]. The incubation period is 2–12 days, and the infectious period in humans is 2–9 days. The life expectancy of mosquitoes is 30 days. Most infected people will have acute symptoms such as high fever, severe joint pain, arthritis, stiffness, and rash. Chronic disease severity varies by age as follows: newborns, young infants, and the elderly are at greater risk for more severe disease. The overall fatality rate is <0.1%, and the disease burden is mainly due to chronic disability and severe impact on patient quality of life [29,30]. Asymptomatic infection rates vary widely across outbreaks and populations, generally ranging from 17% to 39% [31,32]; occasionally, higher rates have been reported [33].

In symptomatic patients, CHIKV disease onset is typically 4–8 days (range 2–12 days) after the bite of an infected mosquito [1,2,34]. It is characterized by an abrupt onset of fever, frequently accompanied by severe joint pain [35,36]. The joint pain is often debilitating and usually lasts for a few days but may be prolonged, lasting for weeks, months or even years [35,37,38]. Persistent arthralgia or chronic inflammatory rheumatic symptoms have been reported in approximately 30–40% of patients [38,39]. Other common signs and symptoms include joint swelling, muscle pain, headache, nausea, fatigue and rash [1,2,34,35,36]. Since these symptoms overlap with other infections, including those with dengue and Zika viruses, cases can be misdiagnosed [1,2,34]. In the absence of significant joint pain, symptoms in infected individuals are usually mild and the infection may go unrecognized [1,2].

Most patients recover fully from the infection; however, occasional cases of eye, heart, and neurological complications have been reported with CHIKV infections [2,35,37]. Reported neurological complications include encephalitis, meningoencephalitis, and Guillain–Barré syndrome [37,40]. Patients at extremes of the age spectrum are at higher risk for severe disease including newborns infected during delivery to infected mothers or bitten by infected mosquitoes in the weeks after birth, and older people with underlying medical conditions. Patients with severe disease require hospitalization because of the risk of organ damage and death [1,2,34]. Once an individual is recovered, available evidence suggests they are likely to be immune from future chikungunya infections [41].

Clinical diagnosis of CHIKV is complicated because its symptoms overlap with other viruses like Dengue, Zika, and MAYV [42,43]. During the first 8 days of illness, reverse transcription-polymerase chain reaction (RT-PCR) on serum samples is the recommended method for detecting CHIKV RNA [42,43,44]. Viral isolation by cell culture may be used and may detect infection during the first 3 days, but it requires experienced personnel, biosafety level 3 laboratories (BSL-3), a long turnaround time, and sensitivity limits that may result in false-negative results in cases of low viral loads [43,45,46]. Typically, near the end of the first week of illness, CHIKV antibodies develop; it will therefore be possible to use tests to detect virus-specific immunoglobulin (Ig) M [42,43]. Positive IgM results should be confirmed by neutralizing-antibody testing because false-positive reactions can occur especially in cases where multiple alphaviruses circulate [42,43,47]. Furthermore, if a sample of the acute phase tests negative but clinical suspicion remains high, it is recommended to test another sample during the convalescent phase [43].

Considering blood transfusions, studies on IgM seroprevalence in highly endemic settings among asymptomatic blood donors can identify recent arbovirus infections and suggest that the donor population may represent a potential source of new infections, particularly during epidemic periods [48]. However, CHIKV seropositivity alone does not establish that a donation is infectious and should complement (rather than replace) the direct detection of viremia [45]. In fact, for transfusion services, nucleic acid amplification testing (NAAT) is most relevant for identifying potentially infectious donations during acute viremia, whereas antibody testing is mainly useful for retrospective diagnosis and epidemiological investigations [49,50]. Currently, there are the following two commercially available, CE-marked NAAT in Europe for screening donors for CHIKV: cobas^®^ CHIKV/DENV test is a real-time duplex RT-PCR test that detects CHIKV and dengue virus serotypes 1–4 in donor plasma [51]; Procleix^®^ Arbo-Plex test is a multiplex transcription-mediated amplification test that detects not only CHIKV but also dengue virus, West Nile virus, and Zika virus in plasma or serum donations [52].

## 4. Recent CHIKV Epidemics and Outbreak Preparedness

**Figure 1:**
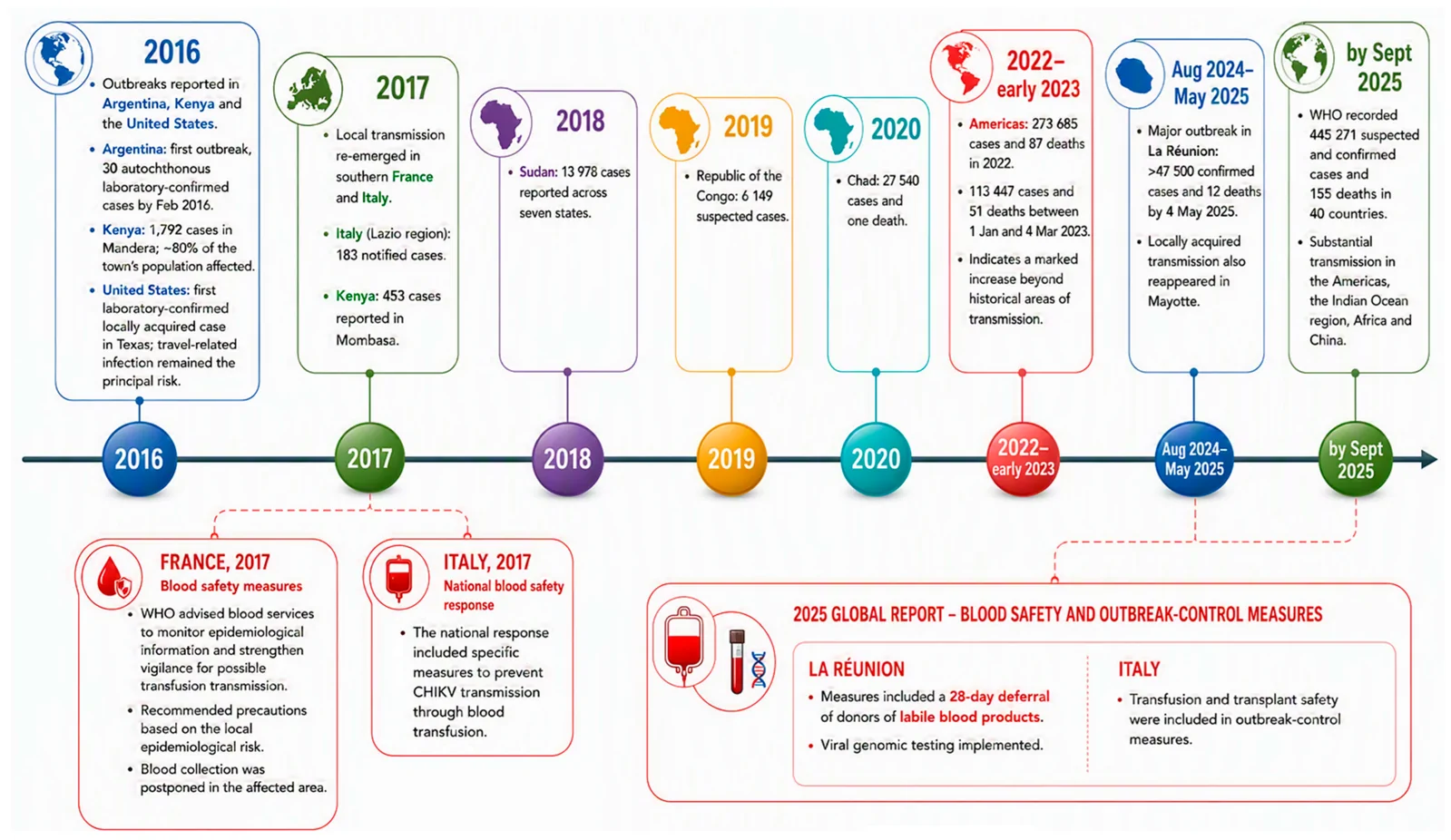
Global CHIKV outbreaks and blood safety interventions reported by WHO over the last 10 years. This figure was generated with the assistance of an AI-based image-generation tool according to the authors’ specifications and was subsequently verified and refined by the authors. No third-party copyrighted images or schematic elements were incorporated.

The epidemics reported by WHO over the last 10 years demonstrate the recurrent and geographically expanding nature of CHIKV transmission. As shown in Figure 1, various outbreaks have been reported around the world as follows: in 2016, outbreaks were reported in Argentina, Kenya and the United States [53,54,55]; in 2017, in southern France and Italy [56,57,58,59]; in 2018, Kenya reported cases in Mombasa, while Sudan recorded cases across seven states [60,61]; in 2019, CHIKV was reported in the Republic of the Congo [62]; in Chad it was also reported in 2020 [63]; between 2022 and early 2023, CHIKV cases and deaths were reported in the Americans [64]; from August 2024 to May 2025, an outbreak affected La Réunion [65]; and lastly, from September 2025, WHO recorded 445,271 suspected and confirmed cases of CHIKV and 155 deaths in 40 countries, with substantial transmission in the Americas, the Indian Ocean region, Africa and China [3].

CHIKV outbreak response requires a One Health approach that includes: individual epidemiological investigation, door-to-door case finding, systematic sampling and lab examination in case of compatible symptoms, destruction of breeding sites, insecticide spraying, information and sensitization of the population, and communication campaigns, especially in non-endemic areas where autochthonous transmission occurs, where public awareness of chikungunya may be limited. In addition, epidemic preparedness should also include measures to protect blood supplies (Figure 1). However, in resource-limited settings, blood screening capacity may be limited by economic and health system resources, which is why it is important to use risk maps that integrate the presence of vectors with ecological and socioeconomic variables that allow us to prioritize surveillance and cost-effective screening in the highest-risk areas [66]. Prevention strategies may include enhanced surveillance of transfusion-transmissible arbovirus infections, pathogen reduction technologies (PRTs), and temporary donor deferral policies, or the use of NAAT on blood donations to ensure blood safety during local outbreaks that could compromise blood supplies [67]. In fact, blood safety was also addressed during the outbreaks in Europe. The WHO recommended increased vigilance to identify any cases of transfusion-transmitted infection, risk-based precautionary measures and, in the affected areas of France, the postponement of blood collections. Italy has implemented measures to prevent transfusion-related transmission, while the response to the 2025 outbreak in Réunion included a 28-day deferral period for blood donors and viral genomic testing (Figure 1). Vaccinating people at highest risk of developing severe forms of disease can further help reduce the public health impact of future outbreaks. From a One Health perspective, entomological surveillance of mosquitoes and molecular research of CHIKV in vectors can help identify viral circulation early. Integrating these data with human case surveillance allows for the rapid activation of vector control and public health measures.

## 5. Risks from Blood Components

CHIKV infection can be highly symptomatic and is associated with high-titre viremia during the acute stage (days 5–10), but up to 40% of infected subjects remain asymptomatic [31]. The viremic levels in asymptomatic CHIKV-infected individuals are also in the range known to be capable of transmitting the disease to experimental animals [45].

The risks of viremic blood donation are related to the viral incidence in the general population and concordant with NAAT results as follows: the risk estimate was 1083/100,000 for CHIKV during an epidemic in French Polynesia [68]. The risk of viremic donations ranged from 38.2 to 52.3 per 100,000 donations during an outbreak in Thailand, and the potential risk of transfusion-associated CHIKF was estimated to be 1 in 1781–2429 donations [69]. In Puerto Rico, CHIKV RNA was detected in 3/557 asymptomatic blood donors during the 2014 epidemic; one donor developed fever and arthralgia two days after donation, whereas the other two remained asymptomatic during follow-up [70]. Another study from the same area shows that donor RNA prevalence reached 1.34% in the epidemic peak months [71]. Some RNA-positive donations contained high viral loads, including values above 10^8^ or 10^9^ copies/mL [70,71], indicating that apparently healthy donors may carry concentrations biologically plausible for transmission.

Blood bags inoculated with CHIKV were able to keep infective viruses during the processing of blood products (red blood cell concentrate, platelet concentrate, and fresh frozen plasma) and also after the recommended storage for each component, which may infect individuals transfused with those [72].

### 5.1. Donor Viremia and Outbreak-Dependent Risk

The frequency of viremic donations is closely linked to the intensity and timing of community transmission. A systematic review and meta-analysis estimated overall CHIKV RNA positivity at approximately 0.5% among blood donors, increasing to about 0.6% in endemic or outbreak settings, falling to zero in non-endemic settings. The highest study-specific RNA-positive rates were reported from Puerto Rico, Thailand, and French Polynesia, at approximately 1.9%, 1.0%, and 1.0%, respectively [73].

Individual outbreak investigations illustrate this heterogeneity. During the 2014 Caribbean epidemic, CHIKV RNA was detected in 0.42% of donations in Guadeloupe and 0.36% in Martinique; however, analyzing donors IgG seroprevalence at the end of the epidemic, the authors found positivity rates of 48.1% and 41.9%, respectively [74]. Earlier outbreak studies reported substantially higher modeled risks during epidemic peaks, including an estimated 1.5% risk of a viremic donation on Réunion Island [75].

The apparent absence of documented clinical transfusion-transmitted CHIKV infection should be interpreted cautiously, since the documentation is limited and it does not prove that transmission is impossible. For example, a systematic review found no confirmed CHIKV transmission through transfusion of blood or blood components, but it also emphasized that arboviral transmission can be difficult to recognize [76]. Recipients may be asymptomatic, investigations may not be performed, and infection acquired from a transfusion can be difficult to distinguish from contemporaneous mosquito-borne exposure during an outbreak [76].

### 5.2. Infectivity in Blood Components

CHIKV can remain infectious in blood, and it has been known since the 1970s that human platelets and platelet aggregates can stabilize viral infectivity [77,78]. Recent study has detected the presence of infectious CHIKV in blood components (red blood cells, platelets and plasma) after they had been subjected to processing and storage conditions typical of transfusion practice [72]. Therefore, it should not be assumed that the routine preparation and storage of blood components inactivate CHIKV, and that the recovery of infectious viruses from an experimental unit demonstrates persistence under the tested conditions, but it does not establish the probability of transmission to a recipient.

A separate discussion is required for plasma-derived medicinal products; in these, it is possible to carry out treatments that effectively eliminate CHIKV, such as heat treatment, treatment with solvents and detergents, and fine-pore nanofiltration [79]. These results confirm the safety of products manufactured using validated viral inactivation processes; however, they must not and cannot be used in the same way for blood components intended for transfusion, as they would lose their functionality.

## 6. Risk Minimization Strategies for Blood Components

In epidemic situations, the large-scale exclusion of donors or the suspension of blood collection may not be sustainable, as it may jeopardize blood supplies. The most appropriate response depends on several factors, such as the intensity and geographical distribution of transmission, the availability of blood components from close unaffected areas, the shelf life of each component, and access to NAAT or PRT. In practice, risk reduction is based on multi-tiered interventions [80].

### 6.1. Donor Selection, Quarantine, and NAAT

Donor questionnaires and symptom-based exclusion are useful but incomplete tools, as infected donors may be asymptomatic or develop symptoms after donation [70,74]. Exclusion based on travel history is particularly relevant when infected donors return to non-endemic regions [81]. Short periods of quarantine can provide an additional safety measure for blood components that can be stored without substantial loss. During the 2017 outbreak in Italy, collection was suspended in the affected areas; red blood cell and plasma units from the surrounding area were quarantined for five days with active donor recall, whilst platelet concentrates underwent PRT [82]. This geographically targeted approach limited disruptions whilst preserving the wider blood supply.

NAAT is capable of identifying donations during the pre-seroconversion window, when the donor questionnaire and antibody tests are less effective. For example, the automated cobas^®^ CHIKV/DENV duplex test has demonstrated high analytical sensitivity for the main CHIKV genotypes and is suitable for high-throughput screening of blood donations [83,84]. A 2024 evaluation of the same test reported a 95% detection limit of 4.76 IU/mL for CHIKV RNA and concluded that the test could ensure blood safety during epidemics [67,83]. However, while NAAT is highly effective, its use remains dependent on test availability and costs, laboratory capacity, turnaround times for results, and the timing of blood collection relative to the short viremic window.

### 6.2. Pathogen-Reduction Technologies

PRT represents a valuable alternative to the expensive NAAT screening for CHIKV RNA, provides broader protection against several pathogens at once, and are useful for labile blood components like platelet concentrates that have short half-life and are difficult to replace through interregional importation during an outbreak. A systematic review [85] identified seven studies that investigated the efficacy of PRTs against CHIKV [86,87,88,89,90,91,92]; these systems are described in Table 1. They measure reduction in infectivity, not simply removal of viral RNA [86,87,88,89,90,91,92].

For example, amotosalen + UVA-light has shown particularly strong laboratory activity against CHIKV. In spiked platelet and plasma units, reductions exceeded 6.40 Log_10_ in platelets and 7.60 Log_10_ in plasma [88]. In platelet plasma, another study reported a reduction greater than 6.52 Log_10_ PFU/mL [92]. Riboflavin-based treatment also achieved complete inactivation under the conditions tested in spiked platelet concentrates, with reductions up to 3.69 Log_10_, although performance can vary by virus and component matrix [90].

PRT also has certain practical limitations as follows: not all systems are available for all components, and its implementation can affect the yield of the components themselves, their storage characteristics, costs and workload. Furthermore, a comprehensive range of PRTs covering all blood components is not yet available everywhere [93]. Consequently, in many blood transfusion systems, PRT may complement NAAT, donor exclusion, quarantine or epidemic surveillance.

### 6.3. High-Dose Irradiation as an Emerging Approach

The routine X-ray irradiation of blood components is carried out at doses of approximately 25 Gy to prevent transfusion-associated graft-versus-host disease; it certainly cannot be assumed that this dose is capable of inactivating CHIKV, should it be present. It should be noted that experimental studies have investigated ultra-high irradiation dose rates (0.1–20 kGy), finding that in platelet preparations contaminated with *Escherichia coli*, doses of 5 kGy or higher inhibited bacterial growth. Therefore, irradiation at ultra-high doses should be investigated as a future broad-spectrum method for reducing bacterial and viral pathogens and as a possible validated alternative to NAAT or established PRTs [94].

## 7. Donor Vaccination

Vaccination may indirectly reduce the risk associated with blood transfusions by preventing CHIKV infection, reducing the number of viremic individuals in the community and reducing the intensity of human-to-mosquito-to-human transmission during outbreaks. Vaccinated individuals may remain susceptible to infection, vaccine-induced immunity may wane, and the relationship between antibody titres and protection against infection has not yet been fully established. The development of the CHIKV vaccine has utilized a wide range of platforms that are thought to be able to balance the strong, durable immune responses often produced by replicating or vector-based vaccines with the safety, manufacturing, and deployment advantages of non-replicating platforms.

Upon analyzing these platforms, it is possible to conclude that, as with other viruses, the main directions for next-generation development are clear because: the goal is to produce non-replicating vaccines that are safer and are used primarily for immunocompromised individuals or pregnant women; the aim is to produce vaccines that can provide a rapid response in the event of an outbreak; there is a desire to produce vaccines that provide broader and longer-lasting immunity, thereby reducing the risk of immune escape; and, of course, vaccines that are easily accessible in terms of cost, formulation, and ease of transport.

### 7.1. Vaccine Pipeline

An updated overview of the vaccine pipeline is presented in Table 2. This pipeline should be interpreted in a temporal context, as several candidates mentioned in previous reviews have been discontinued, suspended, or remain limited to the preclinical research phase [95,96,97].

Currently, candidates that have reached the human trial stage include mRNA-1388, ChAdOx1 Chik, IVI/BBIL BBV87, and MV-CHIK. In 2026, a living systematic review, based on 55 clinical and preclinical studies involving 8279 vaccinated participants, found a high immunogenicity across platforms but greater fever and arthralgia risk with live-attenuated vaccines and limited pregnancy data. This evidence comes from a conference abstract reporting a living review; therefore, they should be interpreted with caution [98].

It is important to highlight that sera generated by candidate vaccines have shown cross-neutralization [99,100]. Specifically, one study tested sera obtained from individuals vaccinated with virus-like particles (VLPs) against nine strains representative of the three main genotypes and found that all nine strains were neutralized [100]. Similarly, sera from subjects who received the VLA1553 vaccine neutralized representative strains from ECSA, West Africa, and Asia; however, the study analyzed a small number of population and measured an in vitro correlate rather than clinical protection [99]. Therefore, these data require confirmation in different populations and, where possible, in studies of protection against infection and viremia in vivo.

### 7.2. Authorized Vaccines and Current Evidence

Since 2023, two vaccines have finally been licensed by both FDA and EMA for subjects >18 years old [101], and their features are summarized in Table 3, but their status and risk–benefit profiles are not interchangeable.

Valneva’s Ixchiq™ is a single-shot, live-attenuated virus (LAV) vaccine based on the La Réunion strain (LR2006-OPY1) of the ECSA genotype [102,103]: attenuation of the LR2006-OPY1 strain was achieved by reverse genetics resulting in a deletion of 61 amino acids within the C-terminal part of the non-structural protein 3 (nsP3) of the replicase complex [102]. This led to smaller plaque size as compared to CHIKV clone LR2006-OPY11 and reduced replication capacity as compared to CHIKV parent clone LR2006-OPY1 in vitro and in vivo [102]: no change in deletion was detectable after serial passages on Vero cells [102]. It has also been approved by the EMA for 12–18 years old subjects. In May 2020, Valneva partnered with Instituto Butantan, Brazil, and in December 2024 with Serum Institute of India for the development, manufacturing (following a tech transfer), and marketing of Valneva’s CHIKV vaccine in low-and-middle income countries. The regulators approved VLA1555 based on the surrogate endpoint “seroresponse rate” in non-human primate models. Protective titer determined in a prospective seroepidemiological trial in the Philippines translated into a microplate plaque reduction neutralization titre (µPRNT50) of ~49 [104], while protection lasts for no less than 3 years [105]. Valneva™ managed an inventory stockpile of up to 200,000 doses, and there is good progress towards expanding the label to <12-years old around 2030. Further expansion will likely be made possible through studies in pregnant women and pediatrics. In 2021 Valneva and Instituto Butantan (IB) signed a Tech Transfer agreement to support affordable supply of VLA1555 in Latin America, and a similar agreement is going to be signed in 2025 with Serum Institute of India (SII) to support affordable supply in Asia. WHO PQ submission of VLA1555 is expected at the end of 2025, while the IB vaccine will be submitted for WHO PQ post-ANVISA licensure. On 7 May 2025, EMA started a review of Ixchiq™ after 17 serious cases have been reported in people from 62 to 89 years of age (including the following two deaths in La Réunion: an 84-year-old man who developed encephalitis, and a 77-year-old man with Parkinson’s disease whose difficulty with swallowing worsened and may have caused aspiration pneumonia) [106].

Bavarian Nordic’s Vimkunya™ is based on VLP. It consists of a prefilled syringe for intramuscular injection with a 3-year shelf-life. Safety, tolerability, and immunogenicity data for Vimkunya™ versus placebo in healthy adolescents and adults aged 12–64 years were investigated in a phase 3 clinical trial (NCT05072080) [107].

### 7.3. Implications for Blood Donors and Transfusion Services

Considering the two vaccines authorized by the EMA and the FDA and their likely effects on donors, certain aspects remain to be clarified. In particular, Vimkunya™, which uses a VLP platform, does not contain a complete infectious genome; therefore, vaccine-associated viremia is not expected. Ixchiq™, which uses attenuated virus technology, can replicate and may cause symptoms like those of chikungunya; it requires careful consideration as it may have contraindications, and attention should also be paid to national guidelines for the exclusion of blood donors. In fact, one should not assume that an attenuated vaccine is irrelevant to blood safety simply because it is designed to be non-pathogenic [95,107]. It must always be kept in mind that, even if donors are vaccinated, they can still contract circulating CHIKV; therefore, vaccination status alone cannot guarantee that a blood donation is virus-free.

Another aspect to consider, as mentioned earlier, is that vaccination coverage of the entire population could reduce susceptibility to circulating CHIKV and consequently indirectly lower the incidence of viremic donations during an outbreak. Vaccination therefore serves as a preventive measure prior to blood component safety measures. There remain some significant gaps in the available evidence as follows: clinical efficacy against symptomatic and asymptomatic infection has not been consistently established; data are limited for the most vulnerable individuals; and the impact of vaccination on donor eligibility or post-vaccination exclusion remains unclear.

## 8. Conclusions and Future Perspective

The literature review and available data suggest that while theoretically possible via blood transfusions, the CHIKV current risk appears very low. Given the short duration of viremia and the transient presence of the virus in peripheral blood, which is usually associated with overt clinical symptoms, donation during the infectious phase is improbable. The possibility of donations from asymptomatic or mildly symptomatic individuals, therefore, merits close consideration of appropriate safety measures in epidemic and endemic settings. It should be emphasized that, although there are no significant numbers of confirmed cases of transfusion-transmitted infection, this does not mean there is no risk [108,109]. Further investigation through prospective studies and dedicated hemovigilance programs would be needed for this purpose [108,110,111]. For this reason, the information collected on other transfusion-transmissible arboviruses, such as West Nile virus, can help operational planning a lot [109,112].

Healthcare systems should strengthen their diagnostic capabilities and reporting systems to quickly identify cases. In fact, studies indicate that during outbreak scenarios, healthcare systems can remain vigilant by using risk mitigation strategies, including temporarily delaying donations from at-risk individuals, applying NAAT, and using PRT [85,113,114].

Climate change, globalization, increasing human mobility, and the ongoing geographic spread of competent vectors will increase CHIKV transfusion transmission even in non-endemic areas now and could drastically alter the epidemiology of CHIKV, which may turn out to be more than just an occasional problem [6,115]. On the other hand, however, the introduction of approved CHIKV vaccines represents a major advance in disease prevention [103,107,116,117,118,119]. It will therefore be essential to assess the potential impact of vaccination on both epidemics and, indirectly, on the risk of viremic donations.

Therefore, improving virological and epidemiological surveillance is vital, as is developing universal, flexible rules that can be quickly modified to fit evolving circumstances [6,120]. Though it does not endanger transfusion safety in most environments, CHIKV is a pathogen that calls for close monitoring as part of an integrated prevention approach to guarantee the continuous safety of blood products.

## Figures and Tables

**Table 1:** Summary of CHIKV RNA mean Log10 reduction factor (LRF) of different PRTs across different blood components.

Blood Components	Riboflavin + UVB Light	Amotosalen + UVA Light	UVC Light (=/- Methylene Blue)	Amustaline (S-303)
fresh frozen plasma	2.10	≥7.60	≥6.58	–
platelets PAS/plasma	2.10	>6.40	6.40	–
platelets in 100% plasma	3.69	5.48	–	–
packed red blood cells or whole blood	–	–	–	6.54

**Table 2:** The pipeline of anti-CHIKV vaccines.

Type of Vaccine	Preclinical	Phase I	Phase IIa	Phase IIb/III
Viral vector	AuroVaccines VSV	U Oxford ChAdOx1	Themis/MSD–MV Hilleman Labs	IVI/BBIL BBV87
	UTMB Eilat Virus			
	Sementia Vaccinia			
	GSK ChAd155			
	Bavarian Nordic MVA			
	CAMS-rVTT-CE1-JE-EGFP			
	VaxArt oral Ad			
RNA	Karolinkska RRP	Moderna mRNA-1388	–	–
	Karolinksa iRNA (infectious)			
	Griffith Univ iRNA (NoLS)			
DNA	Medigen iDNA	–	–	–
	Karolinksa iDNA (infectious DNA)			
	Karolinksa–DREP-env			
Protein-based	Hawaii Biotech rE2	–	–	–
	GSK VLP			
	Texas Tech U VLP multivalent			
	Georgia State U–PapMV rE2EP3			
	Sep Imm Netw E2EP3 peptide			
Inactivated and live attenuated	U North Carolina TMP 17-2	–	–	–
	Takeda IRES			
	Leidos SAVE			
	U Calif–fidelity–variant LAVs			
	Chin Acad of Science LAV delta capsid			
	Sep Imm Netw nsP mutant			
	Indian Imm Formalin 181/25			

**Table 3:** EMA- and FDA-licensed CHIKV vaccines.

Product	Composition	CHIKV Strain	Dosing	Administration/Presentation	Where Licensed	Indicated Age Group	Contraindications
VLA1553 (Ixchiq™, Valneva)	Live attenuated vaccine (LAV)	LR2006-OPY1 (ECSA)	Single dose (104 TCID)	Lyophilized powder, solvent Intramuscular administration (deltoid) 0.5 mL dose	US (November 2023) Canada (June 2024) EU (July 2024) UK (February 2025)	All countries: 18 and above EU: pending label extension to 12 and above	Immunocompromised individuals Caution in pregnant and breastfeeding women
PXVX0317 (Vimkunya™, Bavarian Nordic)	Virus-like particle, aluminum adjuvant	37997 (West African)	20 mg × 2 injections 40 mg × 1 injection	Pre-filled syringe Intramuscular administration 0.8 mL dose	US (February 2025) EU (February 2025)	All countries: 12 and above	Caution in pregnant and breastfeeding women

## Data Availability

No new data were created or analyzed in this study. Data sharing is not applicable to this article.
